# Books and Magazines

**Published:** 1911-01

**Authors:** 


### Books and Magazines.

Some Posological Hints, and Other Useful Information. Issued by The Manufacturers of Fellows' Compound Syrup of Hypophosphites. New York. December, 1910.

This is a handy little book of reference. The most used drugs are arranged alphabetically, and under each heading are given certain *data* relative to its action, absorption and elimination, and the dose of the drug, e. g.: aconite, given by the mouth, begins to act in fifteen minutes. One dose is entirely eliminated within three hours. Should be given every three hours. Present official tincture is three and one-half times weaker than the former; therefore, the dose should be from 5 minims (O. 2 c.c.) to 15 minims. Mr. Fellows, in the preface of the book, says:

"Side by side with the deeper appreciation of the value of extemporaneous prescribing, which has steadily increased during the last decade, the spirit of modern medicine is wholly in favor of the closer study of drugs. A practical knowledge of the rapidity of their absorption and elimination, together with other matters of posological interest is a *sine qua non,* if the best results from their administration are to be expected.

"The value of extemporaneous prescribing is not to be denied, provided the physician who writes his own prescriptions has devoted sufficient time and study to an art which is often grievously neglected in the modern medical school. Text-books, too, are frequently found to be disappointing in their data on the subject of the absorption and elimination of medicines. Such knowledge can only be secured by careful observation and close study, and the experience so gained will not only enable the physician to obtain more satisfactory results, but it will also give him greater confidence in himself, and in the drugs which he will learn *how* to use.

"With these remarks in mind, the following posological hints and information are presented to the profession by The Fellows Company, of New York, in the hope that they may prove of some slight use to the busy practitioner."

A copy may be had on request.

---

Lessons on the Eye.—For the Use of Undergraduate Students. Frank L. Henderson, M. D., ex-President St. Louis Medical Society, 1905; Ophthalmic Surgeon St. Mary's Infirmary; Consulting Oculist to the Wabash Railway, etc. Fourth edition, revised. Philadelphia: P. Blakiston's Sons & Co. 1910. Cloth; price, $1.50.

This manual is well known and very popular. It takes the place of student's class room notes, a bore—rendering note-taking unnecessary, because it contains those things a student would make note of. In the revision, what I may call the "obvious"—say—minute anatomy of the eye,—fitting of glasses, ophthalmoscopy—things an advanced student is supposed to know,—are omitted. It thus makes possible the inserting of things he does not know, within the limits of a student's manual. Get it. The cost is only $1.50.

---

The Principles of Therapeutics. By Dr. A. Manquat, National Correspondent Académie de Médicine. Translated by Dr. M. S. Gabriel. New York and London: D. Appleton & Co., 1910. Cloth, $3.00.

The author dedicates his book to the young physicians who, he says, "will work for a renaissance in therapeutics, which will be the great achievement of twentieth century medicine." That is modest, at least. The *resurrection* of therapeutics is surely a "consummation devoutly to be wished," for it certainly is moribund—if not defunct. Prescription writing is almost a lost art, in these days of ready-made prescriptions, compounded, many of them, in elegant and attractive style. Just why the author looks to the young men to resurrect therapeutics—thus inferentially despairing of the help of the old fellows, I do not know, unless he realizes that it is hard to teach old dogs new tricks. Seriously,—the decadence of therapeutics is due to the neglect of the medical schools to teach it as it should be taught. The student seeing the slight put upon it infers that the professors don't think much of it, anyhow, and they in turn neglect it. It is *fundamental.* The science of therapeutics is the basis of the practice of medicine. Practice without a knowledge of the action, the properties —the effects of drugs—is quackery, pure and simple. Hence this book is *timely,* and most valuable. "Therapeutic nihilism" has become a by-word and a reproach. The Appletons have brought out this work in good shape, and offer it at a very reasonable price. We trust that our readers will catch on,—and let us have a new birth of therepeutics.

